# Distribution and Medical Impact of Loss-of-Function Variants in the Finnish Founder Population

**DOI:** 10.1371/journal.pgen.1004494

**Published:** 2014-07-31

**Authors:** Elaine T. Lim, Peter Würtz, Aki S. Havulinna, Priit Palta, Taru Tukiainen, Karola Rehnström, Tõnu Esko, Reedik Mägi, Michael Inouye, Tuuli Lappalainen, Yingleong Chan, Rany M. Salem, Monkol Lek, Jason Flannick, Xueling Sim, Alisa Manning, Claes Ladenvall, Suzannah Bumpstead, Eija Hämäläinen, Kristiina Aalto, Mikael Maksimow, Marko Salmi, Stefan Blankenberg, Diego Ardissino, Svati Shah, Benjamin Horne, Ruth McPherson, Gerald K. Hovingh, Muredach P. Reilly, Hugh Watkins, Anuj Goel, Martin Farrall, Domenico Girelli, Alex P. Reiner, Nathan O. Stitziel, Sekar Kathiresan, Stacey Gabriel, Jeffrey C. Barrett, Terho Lehtimäki, Markku Laakso, Leif Groop, Jaakko Kaprio, Markus Perola, Mark I. McCarthy, Michael Boehnke, David M. Altshuler, Cecilia M. Lindgren, Joel N. Hirschhorn, Andres Metspalu, Nelson B. Freimer, Tanja Zeller, Sirpa Jalkanen, Seppo Koskinen, Olli Raitakari, Richard Durbin, Daniel G. MacArthur, Veikko Salomaa, Samuli Ripatti, Mark J. Daly, Aarno Palotie, for the Sequencing Initiative Suomi (SISu) Project

**Affiliations:** 1 Analytic and Translational Genetics Unit, Department of Medicine, Massachusetts General Hospital, Boston, Massachusetts, United States of America; 2 Program in Medical and Population Genetics, Broad Institute, Cambridge, Massachusetts, United States of America; 3 Department of Genetics, Harvard Medical School, Boston, Massachusetts, United States of America; 4 Program in Biological and Biomedical Sciences, Harvard Medical School, Boston, Massachusetts, United States of America; 5 Institute for Molecular Medicine Finland, University of Helsinki, Helsinki, Finland; 6 Department of Chronic Disease Prevention, National Institute for Health and Welfare, Helsinki, Finland; 7 Computational Medicine, Institute of Health Sciences, University of Oulu, Oulu, Finland; 8 Wellcome Trust Sanger Institute, Hinxton, Cambridge, United Kingdom; 9 Estonian Genome Center, University of Tartu, Tartu, Estonia; 10 Divisions of Endocrinology and Genetics and Center for Basic and Translational Obesity Research, Children's Hospital Boston, Boston, Massachusetts, United States of America; 11 Medical Systems Biology, Department of Pathology and Department of Microbiology & Immunology, The University of Melbourne, Parkville, Victoria, Australia; 12 Department of Genetics, Stanford University, Stanford, California, United States of America; 13 Stanford Center for Computational, Evolutionary and Human Genomics, Stanford, California, United States of America; 14 Department of Biostatistics and Center for Statistical Genetics, University of Michigan, Ann Arbor, Michigan, United States of America; 15 Lund University Diabetes Center, Department of Clinical Sciences, Diabetes & Endocrinology, Skåne University Hospital, Lund University, Malmö, Sweden; 16 MediCity, University of Turku, Turku, Finland; 17 Department of Medical Microbiology and Immunology, University of Turku and National Institute for Health and Welfare, Turku, Finland; 18 University Heart Centre Hamburg, Clinic for General and Interventional Cardiology, Hamburg, Germany; 19 DZHK (German Centre for Cardiovascular Research), partner site Hamburg/Kiel/Lübeck, Hamburg, Germany; 20 Division of Cardiology, Azienda Ospedaliero-Universitaria di Parma, Parma, Italy; 21 Department of Medicine, Duke University Medical Center, Durham, North Carolina, United States of America; 22 Intermountain Heart Institute, Intermountain Medical Center, Salt Lake City, Utah, United States of America; 23 Division of Cardiology, University of Ottawa Heart Institute, Ottawa, Ontario, Canada; 24 Department of Vascular Medicine, Academic Medical Center, Amsterdam, The Netherlands; 25 Cardiovascular Institute, Perelman School of Medicine at the University of Pennsylvania, Philadelphia, Pennsylvania, United States of America; 26 Division of Cardiovascular Medicine, Radcliffe Department of Medicine, The Wellcome Trust Centre for Human Genetics, University of Oxford, Oxford, United Kingdom; 27 University of Verona School of Medicine, Department of Medicine, Verona, Italy; 28 Department of Epidemiology, University of Washington, Seattle, Washington, United States of America; 29 Cardiovascular Division, Department of Medicine, Washington University School of Medicine, St. Louis, Missouri, United States of America; 30 Center for Human Genetic Research, Massachusetts General Hospital, Boston, Massachusetts, United States of America; 31 Department of Clinical Chemistry, Fimlab Laboratories, University of Tampere School of Medicine, Tampere, Finland; 32 Department of Medicine, University of Eastern Finland, Kuopio, Finland; 33 University of Helsinki, Hjelt Institute, Dept of Public Health, Helsinki, Finland; 34 National Institute for Health and Welfare, Dept of Mental Health and Substance Abuse Services, Helsinki, Finland; 35 Oxford Centre for Diabetes, Endocrinology and Metabolism, University of Oxford, Churchill Hospital, Headington, Oxford, United Kingdom; 36 Wellcome Trust Centre for Human Genetics, University of Oxford, Oxford, United Kingdom; 37 Oxford NIHR Biomedical Research Centre, Churchill Hospital, Headington, Oxford, United Kingdom; 38 Wellcome Trust Centre for Human Genetics, University of Oxford, Oxford, United Kingdom; 39 University of California Los Angeles Center for Neurobehavioral Genetics, Semel Institute for Neuroscience and Human Behavior, University of California Los Angeles, Los Angeles, California, United States of America; 40 Department of Health, Functional Capacity and Welfare, National Institute for Health and Welfare, Helsinki, Finland; 41 Research Centre of Applied and Preventive Cardiovascular Medicine, University of Turku, Turku, Finland; 42 Department of Clinical Physiology and Nuclear Medicine, Turku University Hospital, Turku, Finland; 43 Department of Biometry, Hjelt Institute, University of Helsinki, Helsinki, Finland; 44 Psychiatric & Neurodevelopmental Genetics Unit, Department of Psychiatry, Massachusetts General Hospital, Boston, Massachusetts, United States of America; Emory University, United States of America

## Abstract

Exome sequencing studies in complex diseases are challenged by the allelic heterogeneity, large number and modest effect sizes of associated variants on disease risk and the presence of large numbers of neutral variants, even in phenotypically relevant genes. Isolated populations with recent bottlenecks offer advantages for studying rare variants in complex diseases as they have deleterious variants that are present at higher frequencies as well as a substantial reduction in rare neutral variation. To explore the potential of the Finnish founder population for studying low-frequency (0.5–5%) variants in complex diseases, we compared exome sequence data on 3,000 Finns to the same number of non-Finnish Europeans and discovered that, despite having fewer variable sites overall, the average Finn has more low-frequency loss-of-function variants and complete gene knockouts. We then used several well-characterized Finnish population cohorts to study the phenotypic effects of 83 enriched loss-of-function variants across 60 phenotypes in 36,262 Finns. Using a deep set of quantitative traits collected on these cohorts, we show 5 associations (p<5×10^−8^) including splice variants in *LPA* that lowered plasma lipoprotein(a) levels (P = 1.5×10^−117^). Through accessing the national medical records of these participants, we evaluate the *LPA* finding via Mendelian randomization and confirm that these splice variants confer protection from cardiovascular disease (OR = 0.84, P = 3×10^−4^), demonstrating for the first time the correlation between very low levels of *LPA* in humans with potential therapeutic implications for cardiovascular diseases. More generally, this study articulates substantial advantages for studying the role of rare variation in complex phenotypes in founder populations like the Finns and by combining a unique population genetic history with data from large population cohorts and centralized research access to National Health Registers.

## Author Summary

We explored the coding regions of 3,000 Finnish individuals with 3,000 non-Finnish Europeans (NFEs) using whole-exome sequence data, in order to understand how an individual from a bottlenecked population might differ from an individual from an out-bred population. We provide empirical evidence that there are more rare and low-frequency deleterious alleles in Finns compared to NFEs, such that an average Finn has almost twice as many low-frequency complete knockouts of a gene. As such, we hypothesized that some of these low-frequency loss-of-function variants might have important medical consequences in humans and genotyped 83 of these variants in 36,000 Finns. In doing so, we discovered that completely knocking out the *TSFM* gene might result in inviability or a very severe phenotype in humans and that knocking out the *LPA* gene might confer protection against coronary heart diseases, suggesting that *LPA* is likely to be a good potential therapeutic target.

## Introduction

After widespread success with genome-wide association studies (GWAS) of common variants, several studies have recently begun to identify rare (with <0.5% allele frequency) and low-frequency (0.5–5%) variants in complex diseases and traits such as triglycerides [1], insulin processing [2], bone mineral density [3], Alzheimer's disease [4], impulsivity [5], and prostate cancer [6], some of which confer protection from disease [4]. Protective loss of function variants that can be tolerated in a homozygote state in humans are of particular interest as potential safe targets for therapeutic inhibition. Interestingly, many of these studies that have discovered rare and low-frequency variants use isolated populations that have undergone bottlenecks resulting in frequency enrichment of the associated variants. In contrast to the large number of extremely rare variants present in out-bred populations, such bottlenecked populations have a smaller spectrum of rare variation. This observation has been borne out in examples of Mendelian disease where, for example, Finns and Ashkenazi Jews have characteristic high incidence of recessive diseases because of the enrichment of specific mutations [7], [8], [9] – in the wider European population these same diseases are rarer and have mutational spectra involving a more diverse array of extremely rare mutations. It has not yet been assessed to which extent these population structures, so advantageous to Mendelian studies but of little importance to common variant GWAS, might generally improve the power to identify low-frequency loss-of-function (LoF) variants in studies of complex disease.

To explore this question, we used exome sequencing to characterize the allelic architecture of the Finnish population compared with a set of non-Finnish Europeans (NFEs) from the United States, Great Britain, Germany and Sweden. We demonstrate that Finns carry a significant enrichment of low-frequency (0.5–5%) LoF variation, defined here as nonsense and essential splice sites that are rare in NFEs. In addition to the isolate population structure, Finland has nationwide health records that provide decades of follow-up data that can be linked to epidemiological studies. The availability of nationwide health records in a population isolate structure triggered us to study the impact of low-frequency variants on risk factors and disease outcomes and their risk factors. The Sequencing Initiative Suomi (The SISu project) aims to combine these resources and build knowledge and tools for genome health initiatives. We genotyped 83 LoF variants discovered through our exome sequencing, in several large well-phenotyped population-based cohorts comprised of 36,262 Finns and tested for association to 60 quantitative traits and used data from the 13 disease outcomes assessed using the National Health Registers. We demonstrate that 5 of these variants have significant associations with clinically relevant phenotypes, illustrating the general value of the Finnish population for the study of low-frequency variants studies in complex as well as Mendelian diseases. We further confirm two LoF variants that significantly reduce lipoprotein(a) levels are associated with protection from cardiovascular disease.

## Results

**Figure 1:**
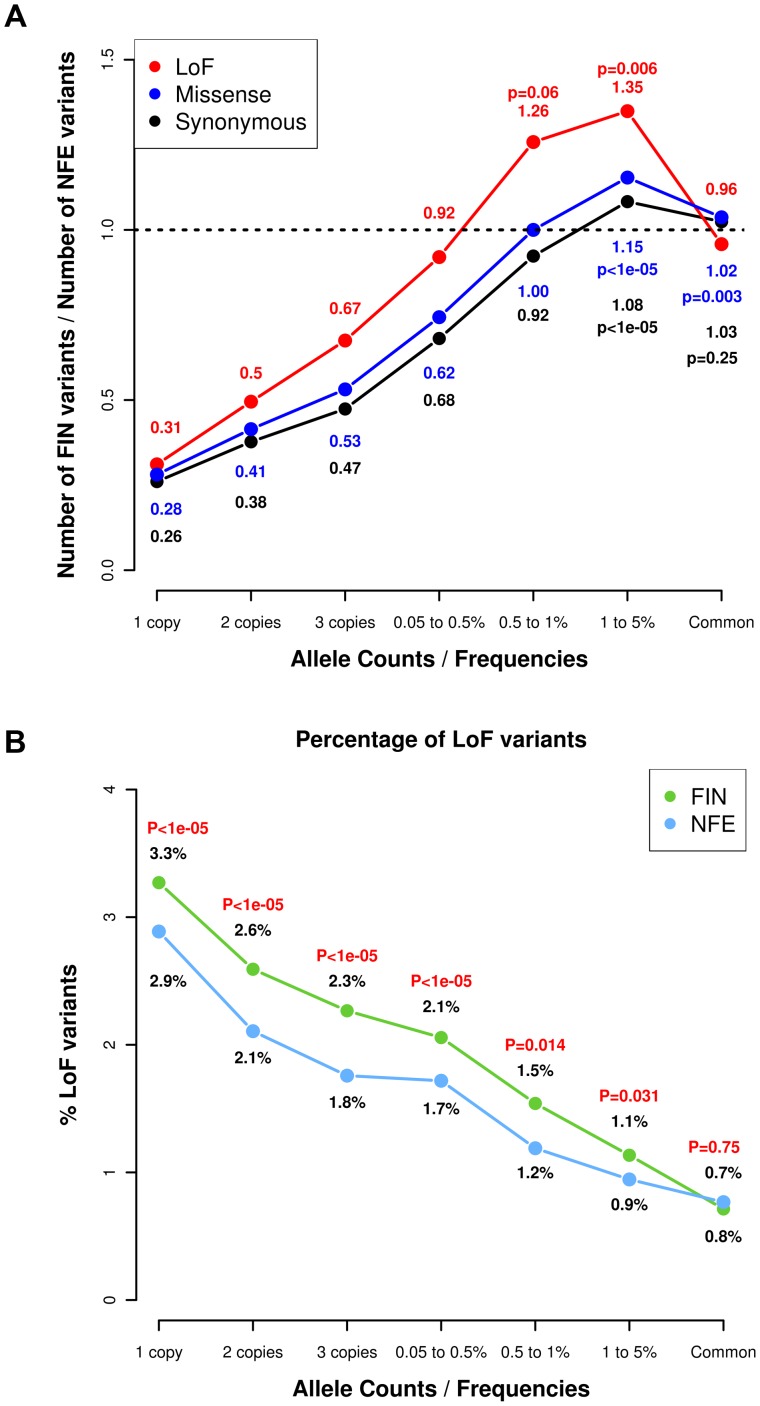
Allele frequency spectrum in Finns and NFEs, demonstrating that Finns have proportionally more deleterious rare and low-frequency variants. (A) Ratio of the number of LoF, missense and synonymous variants found in Finns versus NFEs with the ratios for LoF variants highlighted in red text and the ratios for synonymous variants in black. The p-values represent the probabilities of the excess of variable sites in Finns occurring by chance. The p-values in red represent the probabilities for the LoF variants, the p-values in blue represent the probabilities for the missense variants and the p-values in black represent the probabilities for the synonymous variants. (B) Percentage of variants that are LoF across the allele frequency spectrum, with the numbers indicating the percentage of LoF variants in Finns versus NFEs. The p-values represent the p-values from the hypergeometric test of whether the ratio of LoF variants differ from the ratio of synonymous variants in Finns compared to NFEs.

As part of the SISu Project, we assembled 3,000 whole-exome sequences from Finns in projects including GoT2D, ENGAGE, migraine, METSIM and the 1000 Genomes Project along with 3,000 whole exome-sequences of NFEs from GoT2D, ESP, NIMH and 1000 Genomes project using the same data generation and processing pipelines (Table S1). The raw BAM files from these projects were compressed and re-processed at the Broad Institute and variant calling was performed in a unified manner to minimize potential batch effects. We compared the number and frequency of variable sites in 3,000 Finns and 3000 NFEs (Fig. 1A) and observed several expected hallmarks of the isolated bottlenecked Finnish population history. There was a depletion of ‘singletons’, or variants that were observed only once in 3,000 individuals, in Finns compared to NFEs. An average Finn had 3.7 times fewer singleton variants in these data (binomial P<1×10^−6^). On the other hand, there was an excess of low-frequency variants in Finns versus NFEs (binomial P<1×10^−6^), collectively suggesting that while most rare variants did not survive the bottleneck, the variants that did have become substantially elevated in frequency [10], while the rates of common variation were not different between Finns and NFEs. All these findings are consistent with an expected impact of the Finnish population bottleneck.

**Figure 2:**
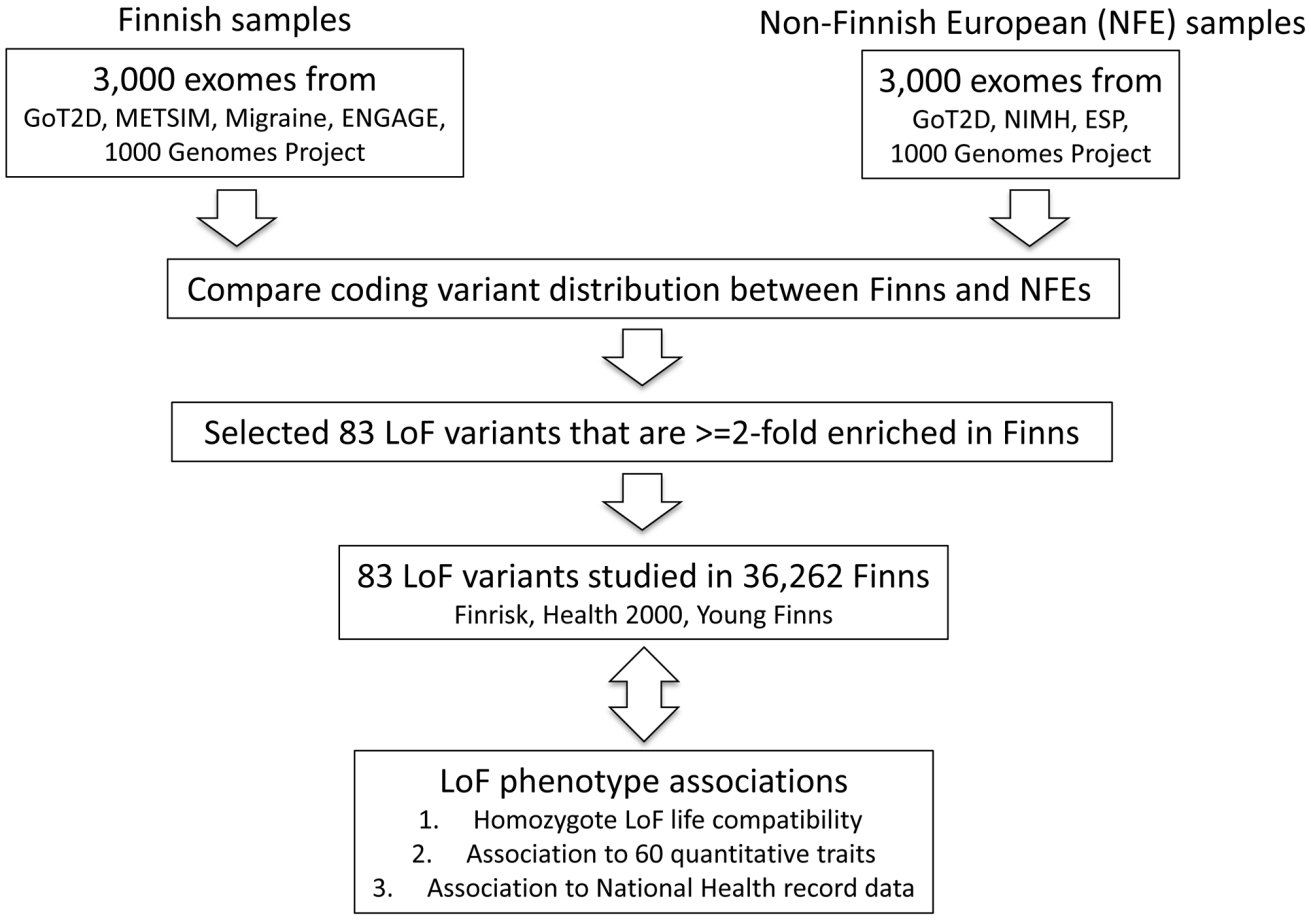
Study design figure for the project. The analysis was performed from an initial set of exome sequences from Finns and NFEs, as well as the selection and survey of the 83 LoF variants across 60 quantitative traits and 13 disease categories.

We then stratified the variants according to their functional annotations – LoF variants, missense variants and synonymous variants. We found a higher proportion of LoF variants in Finns compared to NFEs across the rare and low-frequency allelic spectrum (Fig. 1A, Table S2) and for missense variants predicted to be deleterious by PolyPhen2 (Fig. S1). We found a similar observation when comparing the Finns to an equivalent number of Swedes (Fig. S2). This is also a direct consequence of the bottleneck: alleles that are elevated in frequency through the bottleneck are drawn at random from extremely rare variants in the parental population, where there is a higher proportion of LoF variants that arose recently or were kept at low frequencies because of negative selection. This is clearly demonstrated with the decreasing proportions of LoF variants with increasing allele frequencies (Fig. 1B). The observation that LoF variants in the 0.5–5% range are enriched in Finns and our hypothesis that some of these variants might have health related phenotypic consequences, motivated the targeted association study described below (Fig. 2).

Despite the reduced overall variation in the isolated population, the existence of a greater number of low frequency LoF variants results in an average Finn harboring 0.16 homozygous LoF variants compared to only 0.095 in an average NFE, driven primarily by homozygosity in the 0.5 to 5% allele frequency range (Fig. S3B). These features of the Finnish population have already been well described as they pertain to Mendelian diseases: many characteristic “Finnish founder mutations” exist at unusually high frequencies, even up to 1%, for highly penetrant and reproductively lethal disorders while such variants are extremely rare or absent in NFEs [11]. We confirmed with simulations that while such variants are inevitably pushed to extremely low frequency after 1,000 or more generations, they can easily persist at frequencies between 0.1 and 1% up to 100 generations after a bottleneck (Fig. S4). Table S3 shows a table of a set of Finnish Disease Heritage (www.findis.org) variants and their population frequencies. The extent to which such variants contribute to more common diseases, either through highly-penetrant recessive subtypes or modest risk to carriers, will correspond to advantages in rare and low-frequency association studies in isolated populations.

Given our empirical observations of proportionally more LoF variants in the 0.5–5% allele frequency range in Finns, we next conducted a test of this hypothesis that some of the Finnish-enriched low-frequency LoF variants might have strong phenotypic effects. We successfully genotyped 83 low-frequency LoF variants (protein-truncating nonsense, essential splice site variants and frameshift variants) enriched in Finns based on their ability to multiplex in four Sequenom MALDI-TOF genotyping pools (Table S4). Of these 83 variants, 76 variants were more than 2-fold enriched and 26 were more than 10-fold enriched.in Finns vs. NFEs. Three genes (*SERPINA10*, *LPA* and *FANCM*) contained two LoF variants each; we combined these pairs and tested them as single composite LoF variants, resulting in a total of 80 independent LoF variants tested in this study. These 83 variants were genotyped in a total of 36,262 individuals from three population cohorts: FINRISK [12] (26,245 individuals), Health2000 (7,363 individuals) and Young Finns [13] (2,654 individuals).

As these three studies are population-based cohorts, we were able to assess whether any of the homozygous LoF variants result in such a severe phenotype that these individuals would not be able to participate in a population survey for instance, due to lethality in fetal life of early infancy. Study-wide, there was a modest excess of homozygotes of the variants (1.23-fold versus Hardy-Weinberg expectation) arising from within population substructure. A nonsense variant (Q246X) in the *Translation Elongation Factor, Mitochondrial gene* (*TSFM*) that is present at 1.2% allele frequency in Finns and absent in NFEs, was not found in a homozygous state in >36,000 Finns (Hardy Weinberg Equilibrium (HWE) P = 0.0077). This suggests that complete loss of *TSFM* might result in embryonic lethality, severe childhood diseases in humans, or that the individuals might not have been ascertained by the studies employed, i.e. if the individuals are too sick to be included in the studies. A lookup of this variant in another 25,237 Finnish samples in exome chip genotyping data from the GoT2D studies confirmed that the variant is present at 1.2% in Finns, but again with no homozygotes observed (combined HWE P = 1.6×10^−4^). Recessive missense variants in *TSFM* have been reported to result in mitochondrial translation deficiency [14], [15] and Finnish mitochondrial disease patients from two families have been identified with compound heterozygosity of this nonsense variant (each with a different second hit in *TSFM*) (personal communication) - lending strong evidence to the hypothesis that complete loss of this gene is not tolerated in humans. Neither did we observe strong associations for the *TSFM* Q246X heterozygotes across major diseases (Table S5).

Several other LoF variants occur in genes where recessive mutations have been noted to cause severe Mendelian diseases from the Online Mendelian Inheritance in Man database (OMIM) [16]. For instance, the *Fanconi anemia complementation group M* gene (*FANCM*) was initially discovered in one family with Fanconi anemia [17], but we did not observe any deficit of homozygous LoFs in *FANCM* from our dataset (expected = 5, observed = 7), which we would typically observe for a disease causing recessive variant. Furthermore, examination of the hospital discharge records did not provide any evidence for blood diseases, increased cancer events or any other chronic diseases in these individuals with homozygous LoFs in *FANCM*. We also had blood counts for two homozygote individuals. Both of them had normal hemoglobin, erythrocyte size and counts as well as leukocyte and thrombocyte counts. Singh *et al.* reported that the initial case that led to the association of *FANCM* with Fanconi anemia also harbor biallelic, functional mutations in *FANCA*, a well-established Fanconi anemia gene [18]. Our findings in this study, combined with the findings by Singh *et al.* do not support the hypothesis that *FANCM* is a Fanconi anemia gene but rather suggest that the initial *FANCM* association was not causative. In addition to *FANCM*, we further evaluated evidence for two other genes *COL9A2* and *DPYD* that were previously implicated in other Mendelian diseases (Supplementary Methods).

The FINRISK cohort had collected 60 biochemical and physiological quantitative measurements of cardiovascular or immunologic relevance (Table S6), some of which are highly correlated. We tested the 80 variants across the 60 traits and report from this initial screen all associations with p<2×10^−4^ – that is, a value where we would expect only one chance observation in the entire study. In total, we observed 41 associations that exceeded this significance threshold (Table 1), far beyond the expected. If the phenotype was available in the Young Finns and Health 2000 cohorts, replication was attempted for these initial scan hits and significant associations are highlighted below when the combined p-value was smaller than a conservative study-wide Bonferroni-corrected threshold of 0.05/(80*60) = 1×10^−5^.

**Table 1:** List of top association results from the discovery dataset with p<2×10^−4^.

							Discovery				Replication				Combined (Discovery + Replication)			
Trait	Gene	Chr	Position	Ref	Alt	Allele Freq	N	Beta	SE	P-value	N	Beta	SE	P-value	N	Beta	SE	P-value
**Lp(a)**	***LPA***	**-** *	**-**	**-**	**-**	**0.075**	**6696**	**−0.608**	**0.031**	**2.17×10^−81^**	**2200**	**−0.729**	**0.055**	**6.8×10^−39^**	**8896**	**−0.637**	**0.027**	**1.53×10^−117^**
Vitamin-B12	*FUT2*	19	49206674	G	A	0.62	6087	0.199	0.019	3.68×10^−26^								
**Galectin-3**	***TBPL2***	**14**	**55890937**	**T**	**A**	**0.011**	**6648**	**−0.460**	**0.080**	**9.37×10^−9^**								
GCSF	*ATP2C2*	16	84495318	A	C	0.023	6660	0.272	0.055	6.98×10^−7^	2188	−0.037	0.105	0.73	8848	0.206	0.049	2.2×10^−5^
IL4	*ATP2C2*	16	84495318	A	C	0.023	6660	0.258	0.055	2.48×10^−6^	2188	0.035	0.105	0.74	8846	0.209	0.061	5.91×10^−4^
IFN-gamma	*ATP2C2*	16	84495318	A	C	0.023	6660	0.255	0.055	3.24×10^−6^	2188	0.060	0.105	0.57	8841	0.051	0.016	1.45×10^−3^
IL6	*ATP2C2*	16	84495318	A	C	0.023	6660	0.251	0.055	4.58×10^−6^	2188	0.073	0.105	0.49	8848	0.213	0.049	1.16×10^−5^
Endothelin1	*FUT2*	19	49206674	G	A	0.62	6146	0.086	0.019	5.63×10^−6^								
**D-dimer**	***FGL1***	**8**	**17726470**	**A**	**AT**	**0.037**	**6582**	**0.210**	**0.046**	**6.12E×10^−6^**								
IL12	*ATP2C2*	16	84495318	A	C	0.023	6660	0.245	0.055	8.13×10^−6^	2188	0.042	0.105	0.69	8848	0.201	0.049	3.45×10^−5^
IL17	*ATP2C2*	16	84495318	A	C	0.023	6660	0.241	0.055	1.12×10^−5^	2188	−0.136	0.105	0.20	8848	0.160	0.049	9.91×10^−4^
**Systolic bp**	***ATP2C2***	**16**	**84495318**	**A**	**C**	**0.023**	**25764**	**0.125**	**0.029**	**1.25×10^−5^**	**9355**	**0.113**	**0.054**	**0.037**	**35119**	**0.122**	**0.025**	**1.31×10^−6^**
IFN-gamma	*P4HA3*	11	73978243	G	A	0.32	6655	0.080	0.019	1.70×10^−5^	2186	−0.036	0.032	0.27	8841	0.051	0.016	1.45×10^−3^
IL17	*P4HA3*	11	73978243	G	A	0.32	6655	0.080	0.019	1.72×10^−5^	2186	0.015	0.033	0.63	8841	0.064	0.016	7.27×10^−5^
**Vitamin-B12**	***CLYBL***	**13**	**100518634**	**C**	**T**	**0.035**	**6600**	**−0.203**	**0.047**	**1.83×10^−5^**								
TNF-beta	*HTRA4*	8	38839282	GAA	G	0.013	6669	−0.292	0.069	2.68×10^−5^	2188	0.378	0.141	0.73	8857	−0.172	0.062	5.54×10^−3^
IL4	*ATP10B*	5	160113099	G	A	0.034	6673	0.186	0.045	3.55×10^−5^	2189	0.141	0.086	0.10	8862	0.177	0.040	9.71×10^−6^
FGF	*P4HA3*	11	73978243	G	A	0.32	6655	0.076	0.019	4.58×10^−5^	2186	0.006	0.032	0.85	8841	0.059	0.016	2.81×10^−4^
TNF-beta	*ATP10B*	5	160113099	G	A	0.034	6673	0.184	0.045	4.65×10^−5^	2189	0.091	0.081	0.26	8862	0.164	0.039	3.26×10^−5^
TNF-beta	*ATP2C2*	16	84495318	A	C	0.023	6660	0.223	0.055	4.69×10^−5^	2188	−0.024	0.099	0.81	8848	0.170	0.048	3.86×10^−4^
IFNG	*ATP10B*	5	160113099	G	A	0.034	6673	0.183	0.045	4.81×10^−5^	2189	0.037	0.086	0.67	8862	0.152	0.040	1.45×10^−4^
SDF1	*ATP2C2*	16	84495318	A	C	0.023	6660	0.221	0.055	5.69×10^−5^	2188	0.057	0.105	0.59	8848	0.186	0.049	1.31×10^−4^
TNF-beta	*P4HA3*	11	73978243	G	A	0.32	6655	0.075	0.019	5.94×10^−5^	2186	0.007	0.031	0.81	8841	0.058	0.016	2.66×10^−4^
FGF	*ATP2C2*	16	84495318	A	C	0.023	6660	0.220	0.055	5.98×10^−5^	2188	0.033	0.105	0.75	8848	0.180	0.049	2.11×10^−4^
IL18	*EPPK1*	8	144942134	CCTTT	C	0.023	6677	−0.232	0.058	6.20×10^−5^	2160	−0.240	0.102	0.19	8837	−0.234	0.050	3.42×10^−6^
PDGF	*ATP10B*	5	160113099	G	A	0.034	6673	0.181	0.045	6.20×10^−5^	2189	−0.011	0.086	0.90	8862	0.139	0.040	4.84×10^−4^
SDF1	*ATP10B*	5	160113099	G	A	0.034	6673	0.178	0.045	7.62×10^−5^	2189	−0.118	0.085	0.17	8862	0.114	0.040	4.12×10^−3^
**Triglycerides**	***MS4A2***	**11**	**59863030**	**G**	**A**	**0.019**	**25051**	**0.129**	**0.033**	**7.80×10^−5^**	**9489**	**0.151**	**0.054**	**4.85×10^−3^**	**34540**	**0.135**	**0.028**	**1.31×10^−6^**
VEGF	*HTRA4*	8	38839282	GAA	G	0.013	6669	−0.274	0.069	7.86×10^−5^	2188	−0.010	0.149	0.95	8857	−0.227	0.063	3.10×10^−4^
IL17	*CLYBL*	13	100518634	C	T	0.035	6671	0.185	0.047	8.77×10^−5^								
IL10	*ATP2C2*	16	84495318	A	C	0.023	6660	0.214	0.055	9.33×10^−5^	2188	0.134	0.105	0.20	8848	0.197	0.049	5.08×10^−5^
IL6	*P4HA3*	11	73978243	G	A	0.32	6655	0.072	0.019	9.70×10^−5^	2186	−0.016	0.033	0.62	8841	0.051	0.016	1.73×10^−3^
IL17	*HTRA4*	8	38839282	GAA	G	0.013	6669	−0.270	0.069	1.03×10^−4^	2188	−0.008	0.149	0.96	8857	−0.223	0.063	3.97×10^−4^
IFN-gamma	*CCL26*	7	75401263	C	T	0.013	6663	−0.307	0.080	1.15×10^−4^	2192	−0.183	0.133	0.17	8855	−0.274	0.068	5.94×10^−5^
IL12	*ATP10B*	5	160113099	G	A	0.034	6673	0.174	0.045	1.20×10^−4^	2189	0.010	0.086	0.91	8862	0.138	0.040	5.41×10^−4^
IL6	*ATP10B*	5	160113099	G	A	0.034	6673	0.173	0.045	1.23×10^−4^	2189	0.024	0.086	0.78	8862	0.141	0.040	4.15×10^−4^
IL17	*ATP10B*	5	160113099	G	A	0.034	6673	0.173	0.045	1.24×10^−4^	2189	−0.003	0.086	0.97	8862	0.135	0.040	7.13×10^−4^
PDGF	*P4HA3*	11	73978243	G	A	0.32	6655	0.071	0.019	1.31×10^−4^	2186	−0.022	0.032	0.50	8841	0.048	0.016	2.86×10^−3^
GCSF	*CLYBL*	13	100518634	C	T	0.035	6671	0.180	0.047	1.41×10^−4^								
PDGF	*ATP2C2*	16	84495318	A	C	0.023	6660	0.209	0.055	1.43×10^−4^	2188	0.048	0.105	0.65	8848	0.174	0.049	3.40×10^−4^
GCSF	*EFCAB3*	17	60469326	C	T	0.043	6606	0.157	0.042	1.86×10^−4^	2192	−0.157	0.079	0.046	8798	0.087	0.037	0.018

The associations that are experiment-wide significant highlighted in bold. The discovery dataset is a subset from FINRISK and the replication datasets are from the Health2000 and Young Finns studies.

* The 2 splice variants in *LPA* were combined to obtain a composite *LPA* variant for the association analyses.

Three of these association have been previously reported and represent positive controls for our approach: a strong association for the 2 splice variants (c.4974-2A>G and c.4289+1G>A) in the *Lipoprotein(a)* gene (*LPA*) with lipoprotein(a) measurements in plasma (P_discovery_ = 2.17×10^−81^, P_discovery+replication_ = 1.53×10^−117^, combined 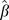 = −0.64 or −8.77 mg/dL per allele, Table S7), the W154X variant in *Fucosyltransferase 2* (*FUT2*) with increased Vitamin B12 levels [19] (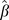 = 0.2, P = 3.7×10^−26^ or 43 pg/mL per allele, Table S8) and the R225X variant in the *Citrate Lyase Beta Like* gene (*CLYBL*) with decreased Vitamin B12 levels [20] (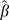 = −0.2, P = 1.8×10^−5^ or −43 pg/mL per allele, Table S9) [21]. The boxplots for these associations are shown in Fig. S5.

In addition to a strong correlation between circulating lipoprotein(a) levels and cardiovascular disease, it has been previously reported that genetic variants that elevate circulating lipoprotein(a) levels are cardiovascular risk factors [22], [23]. The converse, critical for evaluation of the therapeutic hypothesis of inhibition, that lowering lipoprotein(a) levels can confer cardiovascular protection has not yet been evaluated. With access to National Health Records, we utilized the strong lipoprotein(a) lowering variants discovered here to evaluate the impact of lipoprotein(a) lowering via Mendelian randomization. Using a Cox proportional hazards model for incident cardiovascular disease in these cohorts (adjusted for age, gender and therapies), the composite *LPA* variant was found to protect against coronary heart disease (Hazard Ratio HR = 0.79, P = 6.7×10^−3^), demonstrating that lowering lipoprotein(a) levels are likely to confer protection for cardiovascular diseases. We adjusted the association for the composite *LPA* variant with a previously published risk variant (rs3798220) [22], but observed a similarly protective effect (N = 18,270, HR = 0.79, P = 0.014), suggesting that the splice variants are independent from the previously reported risk variants in *LPA*.

**Figure 3:**
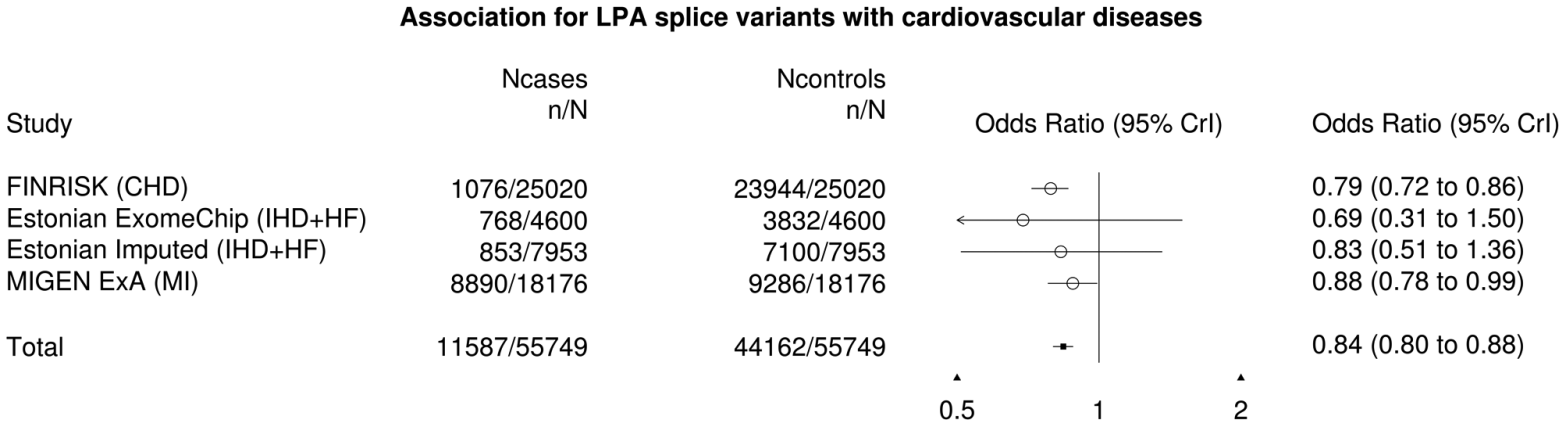
Forest plot for the *LPA* splice variants with cardiovascular diseases. The cardiovascular diseases were defined as coronary heart disease (CHD), ischemic heart disease (IHD), heart failure (HF) or myocardial infarction (MI) from the various cohorts.

We confirmed this finding using three independent non-Finnish datasets: an early onset myocardial infarction dataset of 18,000 individuals and two studies from the Estonian Biobank (4,600 and 7,953 individuals respectively), which collectively replicated the observation that the *LPA* variants confer cardioprotective effect (OR = 0.87, P = 0.016). After meta-analyzing all the datasets, the final odds ratio was found to be 0.84 (P = 3×10^−4^, Fig. 3). We found 227 individuals who are homozygous or compound heterozygous for the two *LPA* splice variants with no evidence for increased morbidity or mortality based on National Health Records. This suggests that reduction of lipoprotein(a) is well-tolerated and might constitute a potential drug target for cardiovascular diseases. A survey across other diseases showed potential association between the *LPA* variants with acute coronary disease and myocardial infarction but not Type 2 Diabetes (Table S10). In addition, we surveyed the *LPA* variants across other cardiovascular risk factors and observed that the *LPA* variants were associated with mildly increased glucose levels but not high-density lipoproteins (HDL), low-density lipoproteins (LDL) or triglycerides (Table S11).

In addition, we observed novel associations for the *FGL1*, *MS4A2* and *ATP2C2* variants. The 1-bp c.545_546insA frameshift in the *Fibrinogen-like 1* gene (*FGL1*) was associated with increased D-dimer levels (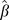 = 0.21, P = 6.1×10^−6^ or 52.23 ng/mL per allele, Table S12). D-dimers are products of fibrin degradation and their concentration in the blood flow is clinically used to monitor thrombotic activity. The role of *FGL1* in clot formation remains unclear: although *FGL1* is homologous with fibrinogen, it lacks the essential structures for fibrin formation, with one study suggesting its presence in fibrin clots [24]. In addition, given prior links between variants associated with D-dimer levels and stroke, we utilized the same Mendelian randomization approach as for *LPA* above and found a nominally significant association between *FGL1* c.545_546insA and increased risk of ischemic stroke (OR = 1.32, P = 0.024). If replicated, this would be consistent with modest risk increase for stroke that other variants associated to circulating D-dimer levels, such as reported for variants in coagulation *Factor V*, *Factor III* and *FGA* [25].

We found suggestive associations for the c.637-1G>A splice variant in the *membrane-spanning 4-domains, subfamily A, member 2* gene (*MS4A2*) with triglycerides (P_discovery_ = 7.80×10^−5^, P_discovery+replication_ = 1.31×10^−6^, 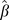 = 0.14 or 0.14 mmol/L per allele, Table S13). This observation is consistent with our previously published study of 631 individuals in the DILGOM subset of FINRISK showing that whole blood expression of *MS4A2* was strongly negatively associated with total triglycerides (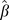 = −1.62, P = 2.1×10^−27^, Fig. S6) [26] and a wide range of systemic metabolic traits [27]. A similar but insignificant trend was observed in 15,696 individuals from the D2D2007, DPS, FUSION, METSIM and DRSEXTRA cohorts (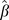 = 0.04, P = 0.32). The *MS4A2* gene encodes the β-subunit of the high affinity IgE receptor, a key mediator of the acute phase inflammatory response.

The c.2482-2A>C splice variant in the *ATPase Ca++ Transporting Type 2C Member 2* gene (*ATP2C2*) was associated with increased systolic blood pressure (P_discovery_ = 1.25×10^−5^, P_discovery+replication_ = 1.3×10^−6^, 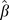 = 0.12 or 2.13 mmHg per allele (an association that is undisturbed by correction for lipid lowering medication (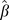 = 0.12, P = 1.75×10^−5^) or blood pressure lowering medication (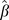 = 0.13, P = 1.3×10^−5^), Table S14). Based on its structure, *ATP2C2* is predicted to catalyze the hydrolysis of ATP coupled with calcium transport. Interestingly, the *ATP2C2* c.2482-2A>C variant is also significantly associated to several highly correlated immune markers, such as granulocyte colony-stimulating factor (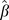 = 0.26, P = 6.98×10^−7^), interleukin-4 (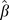 = 0.27, P = 2.48×10^−6^), interferon-γ (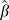 = 0.26, P = 3.24×10^−6^) and interleukin-6 (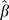 = 0.25, P = 4.58×10^−6^).

## Discussion

The empirical data of this study sheds light on an active debate in population genetics theory whether or not bottlenecked populations have an excess burden of deleterious alleles. Lohmueller *et al.* first observed that there were proportionally more deleterious variants in European American individuals compared to African American individuals [28]. They performed a series of forward simulations to demonstrate that such an observation is consistent with an Out-of-Africa bottleneck experienced by the European populations from which the European-American individuals descend, and illustrated that bottlenecked populations are likely to accumulate a higher proportion of deleterious alleles. A recent study by Simons *et al.* showed conflicting results suggesting that there are similar burdens of deleterious alleles in Europeans and West Africans and that demography is unlikely to contribute to the proportions of deleterious alleles in human populations [29].

The comparison of Finns, with a well-documented bottleneck, with non-Finnish Europeans here provides strong empirical data on these questions. While the distribution of common alleles, both synonymous and non-synonymous, is as expected unchanged by the bottleneck, when exploring the rare and low-frequency allelic spectrum where the Finns and NFEs demonstrate distinct distributions, we indeed observe a significant excess of deleterious variants in the Finns – despite the considerable deficit in variable sites in the population overall. This suggests that negative selection has had insufficient time to suppress the frequency of deleterious alleles dramatically elevated in frequency through the founding bottleneck, an observation that generalizes the intuitive understanding of the existence of characteristic and unusually common Mendelian recessive disorders in Finland. However, we note that while we observe a strong influence of the founding bottleneck, the observed results, particularly the proportional enrichment of rare deleterious variants, are also influenced by other elements in the unique history of the Finnish population and will not necessarily apply to all populations influenced by a bottleneck.

This excess of presumably deleterious variants motivated the subsequent association study and indeed, the absence of homozygotes at *TSFM* (contemporaneously identified as an early-onset mitochondrial disease gene) suggests that low-frequency variants in Finns, beyond those already identified in Mendelian disease, do include more unusually strong acting alleles than in non-founder populations. In this study, both replicated results and novel associations demonstrate the association of low-frequency LoF variants with various complex traits and diseases. In addition, we discovered a novel cardiovascular protective effect from splice variants in the *LPA* gene, suggesting that knocking down levels of circulating lipoprotein(a), or Lp(a), can confer a protection from cardiovascular diseases. Given that we detected numerous individuals in these adult population cohorts, healthy and in the expected Hardy-Weinberg proportions, carrying a complete knockout of *LPA* (homozygous or compound heterozygous for the 2 splice variants), this suggests that knocking out the gene in humans does not result in severe medical consequences. As such, this study provides data suggesting that *LPA* may be an effective target for therapeutic purposes.

As more Finnish samples are being sequenced, these enriched variants can also be imputed with high precision to the large number of existing samples with array-based GWAS genotypes. This advantage is likely to be more pronounced for the much larger pool of missense variation – while one can presume all LoF variants in a gene might have a comparable effect on phenotype (and thereby burden tests of LoF variants in an out-bred sample is not at a great disadvantage compared to isolated populations), it is evident that many rare missense variants within the same gene will not all have the same impact on gene function. Thus the ability to assess single low-frequency variants conclusively, especially since they will include an excess of damaging variants enriched through a bottleneck, rather than perform burden tests on heterogeneous sets of extremely rare variants, will offer substantial ongoing advantage to isolated population studies as indicated by these and other recent findings.

## Materials and Methods

All research involving human participants have been approved by the Hospital District of Helsinki and Uusimaa Coordinating Ethical Committee, and all clinical investigation was conducted according to the principles expressed in the Declaration of Helsinki.

### Exome sequencing quality control, annotation and filtering

Raw Binary Sequence Alignment/Map (BAM) files from the various projects were jointly processed at the Broad Institute and joint variant calling was performed on all exomes to minimize batch differences. Functional annotation was performed using the Variant Effect Predictor (VEP v2.5) tool from Ensembl (http://useast.ensembl.org/info/docs/tools/vep/). We modified it to produce custom annotation tags and additional loss-of-function annotations. The additional annotations were applied to variants that were annotated as STOP_GAINED, SPLICE_DONOR_VARIANT, SPLICE_ACCEPTOR_VARIANT, and FRAME_SHIFT and the variants were flagged if any filters failed. A loss-of-function variant was predicted as high confidence if there is one transcript that passes all filters, otherwise it is predicted as low confidence. In our genotyping study, we had used loss-of-function variants that were predicted to be high confidence. For quality control, we required all variants to pass the basic GATK filters and required all genotypes to have a quality score of ≥30, read depth of ≥10 and allele balance of between 0.3 and 0.7 for heterozygous calls and <0.1 for homozygous calls. Allele counts and frequencies were calculated within the 3,000 individuals for Finns and NFEs respectively.

### Detecting amount of substructure in the Finnish and NFE exomes

To estimate the amount of substructure or homozygosity by descent, we fitted a regression model on all coding variants with the intercept set to 0, where *q* is the allele frequency of the alternate allele and *F_ST_* is the proportion of allelic variance explained by population structure. Here we fit *F_ST_* to capture the empirical departure from Hardy-Weinberg equilibrium arising from population substructure to insure this is not creating the observed difference between Finnish and NFE samples:
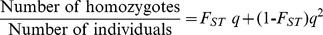
Using the whole-exome sequencing data for the 3,000 NFEs, we estimated the parameters:
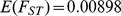


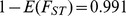


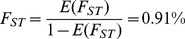
Using the whole-exome sequencing data for the 3,000 Finns, we estimated the parameters:
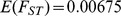


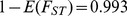


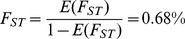
As shown, there is little substructure in the 3,000 Finns compared to the 3,000 NFEs, given that the estimates for *F_ST_* are similar in both populations.

### Variant selection for genotyping

All frameshifts and loss-of-function single nucleotide variants with allele frequencies of 0.5–5% in Finns and at least 2-fold enriched in Finns compared to NFEs were selected for genotyping. To minimize the false positives in our variant selection, we performed Fisher's Exact Test for each variant between two independent NFE datasets and kept variants whose allele frequencies were highly concordant between the two NFE datasets (P>1×10^−5^). The high concordance between the allele frequencies in two independent NFE datasets ensures that the variants are unlikely to arise from alignment or sequencing artifacts and that these variants are unlikely to reside in a region of the exome that is difficult to sequence or genotype, which can result in highly variable allele frequencies from different experiments.

### Sequenom genotyping

Genotyping was performed using the iPLEX Gold Assay (Sequenom Inc.). Assays for all SNPs were designed using the eXTEND suite and MassARRAY Assay Design software version 3.1 (Sequenom Inc.). Amplification was performed in a total volume of 5 µL containing ∼10 ng genomic DNA, 100 nM of each PCR primer, 500 µM of each dNTP, 1.25× PCR buffer (Qiagen), 1.625 mM MgCl_2_ and 1 U HotStar Taq (Qiagen). Reactions were heated to 94°C for 15 min followed by 45 cycles at 94°C for 20 s, 56°C for 30 s and 72°C for 1 min, then a final extension at 72°C for 3 min. Unincorporated dNTPs were SAP digested prior to iPLEX Gold allele specific extension with mass-modified ddNTPs using an iPLEX Gold reagent kit (Sequenom Inc.). SAP digestion and extension were performed according to the manufacturer's instructions with reaction extension primer concentrations adjusted to between 0.7–1.8 µM, dependent upon primer mass. Extension products were desalted and dispensed onto a SpectroCHIP using a MassARRAY Nanodispenser prior to MALDI-TOF analysis with a MassARRAY Analyzer Compact mass spectrometer. Genotypes were automatically assigned and manually confirmed using MassARRAY TyperAnalyzer software version 4.0 (Sequenom Inc.). The genotyped variants were then checked for concordance in allele frequencies with the exome sequencing data.

### Phenotyping

Data on disease status from National Health registers (Hospital Discharged Registers maintained by THL (Institute for Health and Welfare, Finland), Cause of Death Register, Statistics Finland and Prescription Medication Register, THL) for FINRISK, Health2000 and the Young Finns Study participants of this study were collected and curated. A description of each cohort is provided in the Supplement.

### Analyses of RNA sequencing data

To analyze the effects of the LoF variants on gene expression, we used RNA sequencing data from two major studies: the GEUVADIS project [30] with RNA sequencing data from lymphoblastoid cell lines of 462 individuals participants from the 1000 Genomes Project [31]), and the GTEx project with RNA-sequencing data from a total of 175 individuals with 1–30 tissues each (http://www.broadinstitute.org/gtex/) [32]. The processing of the GEUVADIS data and the methods for allele-specific expression analysis are described in Lappalainen *et al.* [30] and the GTEx data were analyzed using similar methods. Allele-specific expression analysis was used primarily to capture nonsense-mediated decay. Additionally, to assess whether LoF variants lead to decreased exon expression levels overall or for individual exons, we calculated an empirical p-value for each exon of all the LoF genes with respect to all other exons genome-wide, denoting the proportion of all exons where carriers of the LoF variants are more extreme than in the each studied exon in LoF variant genes. The analyses were performed separately in each studied tissue: lymphoblastoid cell lines from the GEUVADIS data and nine tissues from the GTEx data. The significance threshold after correcting for the total number of tested exons across all tissues is 0.05/1070 = 4.67×10^−5^.

### Statistical analyses and methods

Inverse rank-based normalization was performed on the quantitative measurements in males and females separately, with linear regression residuals using age and age^2^ as covariates. Linear regression was then performed on the normalized Z-scores using R to obtain the statistics for the associations. We tested the correlations between the quantitative measurements and disease outcomes using two one-tailed t-tests to assess the significance of observing higher levels of the quantitative measurements in cases (individuals with the disease outcomes) versus controls (individuals without the disease outcomes), as well as lower levels of the quantitative measurements in cases versus controls. To test the association of the variants with the prevalent disease outcomes, we performed a logistic regression in R to obtain the reported statistics. In addition, a Fisher's Exact Test on the homozygous counts in cases and controls were performed to test for association with the homozygotes. The results for the LPA with cardiovascular disease association from MIGen ExA and the Estonian Biobank were meta-analyzed using METAL [33] and the combined results with FINRISK were obtained using the Fisher's Combined P method with 4 degrees of freedom.

### Associations between *MS4A2* c.637-1G>A, gene expression and triglycerides

We fit a linear model in which the log_2_-normalised gene probe expression of individual *i* was regressed on the LoF genotype, which was encoded as *X_i_* = 0, 1 or 2 for the LoF genotypes −/−, +/− or +/+ respectively and association analysis of *MS4A2* gene expression and triglycerides was performed as previously reported [26]. Briefly, we used a multivariate linear regression adjusted for age, gender, and use of cholesterol or blood pressure lowering medication. We further tested for association between *MS4A2* c.637-1G>A and triglycerides using a 2-sided t-test.

## Supporting Information

Figure S1 Ratio of the number of missense variants predicted by PolyPhen2 found in Finns versus NFEs. (A) The ratios for probably damaging missense variants highlighted in red text and the ratios for benign missense variants in black. The p-values represent the binomial probabilities of the variants being enriched in Finns and similarly, the p-values in red represent the probabilities for the probably damaging missense variants and the p-values in black represent the probabilities for the benign missense variants. (B) Percentage of variants that are missense variants across the allele frequency spectrum.
(DOCX)

Figure S2 Allele frequency distribution in 3,000 Finns compared to 3,000 Swedes. The ratios for LoF variants highlighted in red text and the ratios for synonymous variants in black.
(DOCX)

Figure S3 Distribution of LoF variants per individual. (A) Number of LoF variants in an average Finn vs NFE individual. (B) Number of homozygous LoF variants in Finns vs NFEs per individual.
(DOCX)

Figure S4 Simulations for a set of variants (ranging from 1% to 5% allele frequencies) with complete recessive lethality. The red line indicates the expected allele frequencies in present-day Finns (where the Finnish bottleneck occurred ∼100 generations ago) and the blue line indicates the expected allele frequencies in Finns 1,000 generations after the Finnish bottleneck, similar to the out-of-Africa bottleneck which occurred >1,000 generations ago.
(DOCX)

Figure S5 Boxplots for the known and novel associations.
(DOCX)

Figure S6 Correlation between triglycerides and *MS4A2* gene expression.
(DOCX)

Table S1 Exomes collected from ongoing studies. All the Finnish and NFE exome sequences were captured using the Agilent SureSelect v2 kit. The replication data for the *LPA* variants from the different studies was performed on the exome chip genotyping platform.
(XLSX)

Table S2 The number of variants in each category in Finns and NFEs.
(XLSX)

Table S3 Allele frequencies of variants discovered from the FinDis database.
(XLSX)

Table S4 Final list of variants from Sequenom genotyping in 36,262 Finns. The cohorts used in this study are from FINRISK 1992, FINRISK 1997, FINRISK 2002, FINRISK 2007, Health 2000 and Young Finns studies (83 variants + 3 composite variants).
(XLSX)

Table S5 Associations between *TSFM* Q246X heterozygotes and various disease states, as well as various neurological and muscular diseases from the medical record system (ICD 9/10) with >30 cases.
(XLSX)

Table S6 List of 60 blood pressure measures and biochemical assays from plasma/serum of fasting subjects.
(XLSX)

Table S7 Correlations between the combined *LPA* variant and various disease states. The rows with significant correlation between the levels of the biomarker and disease status (P<1×10^−3^) are shaded in blue and the rows with significant association (P≤0.05) between the variant and disease status (allelic or homozygous tests) are highlighted in red text.
(XLSX)

Table S8 Correlations between *FUT2* W154X and various disease states. The rows with significant correlation between the levels of the biomarker and disease status (P<1×10^−3^) are shaded in blue and the rows with significant association (P≤0.05) between the variant and disease status (allelic or homozygous tests) are highlighted in red text.
(XLSX)

Table S9 Correlations between *CLYBL* R225X and various disease states. The rows with significant correlation between the levels of the biomarker and disease status (P<1×10^−3^) are shaded in blue and the rows with significant association (P≤0.05) between the variant and disease status (allelic or homozygous tests) are highlighted in red text.
(XLSX)

Table S10 Association of the composite *LPA* variant with different diseases.
(XLSX)

Table S11 Association of *LPA* composite variant with other potential cardiovascular risk factors.
(XLSX)

Table S12 Correlations between *FGL1* c.545_546insA and various disease states. The rows with significant correlation between the levels of the biomarker and disease status (P<1×10^−3^) are shaded in blue and the rows with significant association (P≤0.05) between the variant and disease status (allelic or homozygous tests) are highlighted in red text.
(XLSX)

Table S13 Correlations between *MS4A2* c.637-1G>A and various disease states. The rows with significant correlation between the levels of the biomarker and disease status (P<1×10^−3^) are shaded in blue and the rows with significant association (P≤0.05) between the variant and disease status (allelic or homozygous tests) are highlighted in red text.
(XLSX)

Table S14 Correlations between *ATP2C2* c.2482-2A>C and various disease states. The rows with significant correlation between the levels of the biomarker and disease status (P<1×10^−3^) are shaded in blue and the rows with significant association (P≤0.05) between the variant and disease status (allelic or homozygous tests) are highlighted in red text.
(XLSX)
