# Supplementary figures and images for: Distribution and Medical Impact of Loss-of-Function Variants in the Finnish Founder Population
Source: PLoS Genet. 2014 Jul 31;10(7):e1004494. doi: 10.1371/journal.pgen.1004494 (PMC4117444; doi:10.1371/journal.pgen.1004494)

Figure S1


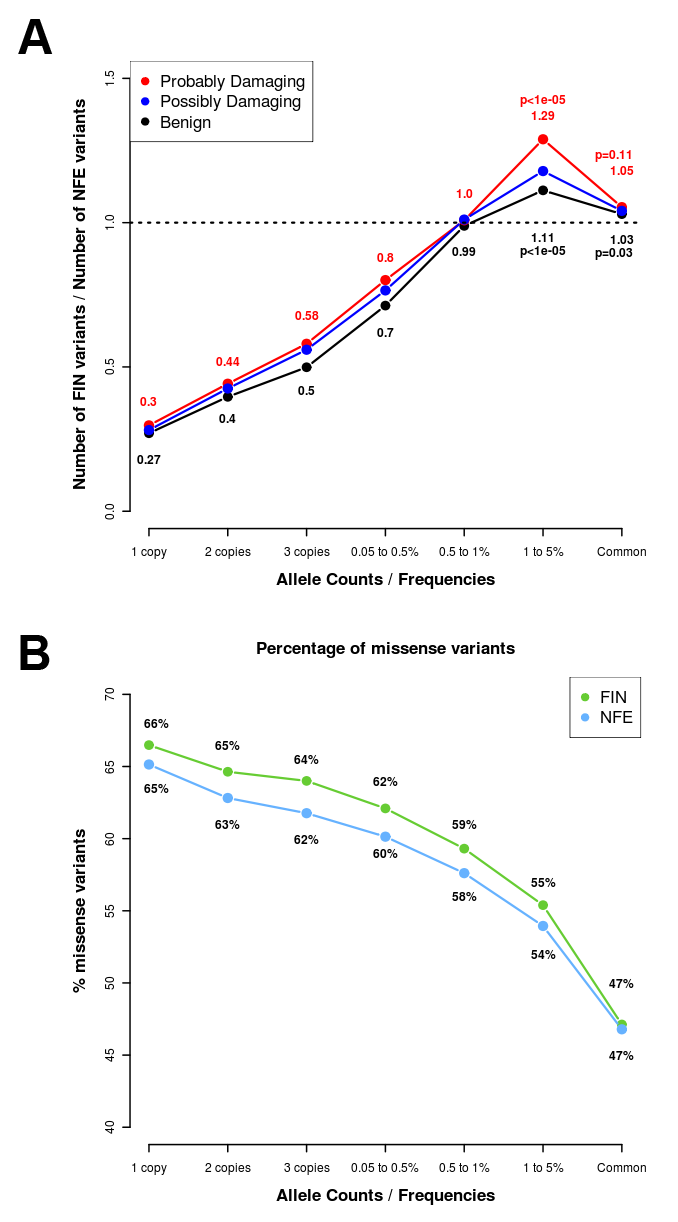

Supplement: Figure S1 — Ratio of the number of missense variants predicted by PolyPhen2 found in Finns versus NFEs. (A) The ratios for probably damaging missense variants highlighted in red text and the ratios for benign missense variants in black. The p-values represent the binomial probabilities of the variants being enriched in Finns and similarly, the p-values in red represent the probabilities for the probably damaging missense variants and the p-values in black represent the probabilities for the benign missense variants. (B) Percentage of variants that are missense variants across the allele frequency spectrum. (DOCX) [file pgen.1004494.s001.docx]

Figure S2


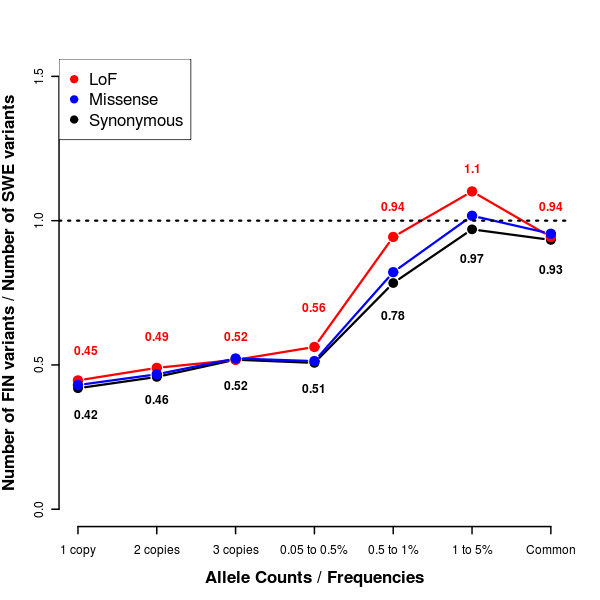

Supplement: Figure S2 — Allele frequency distribution in 3,000 Finns compared to 3,000 Swedes. The ratios for LoF variants highlighted in red text and the ratios for synonymous variants in black. (DOCX) [file pgen.1004494.s002.docx]

Figure S3


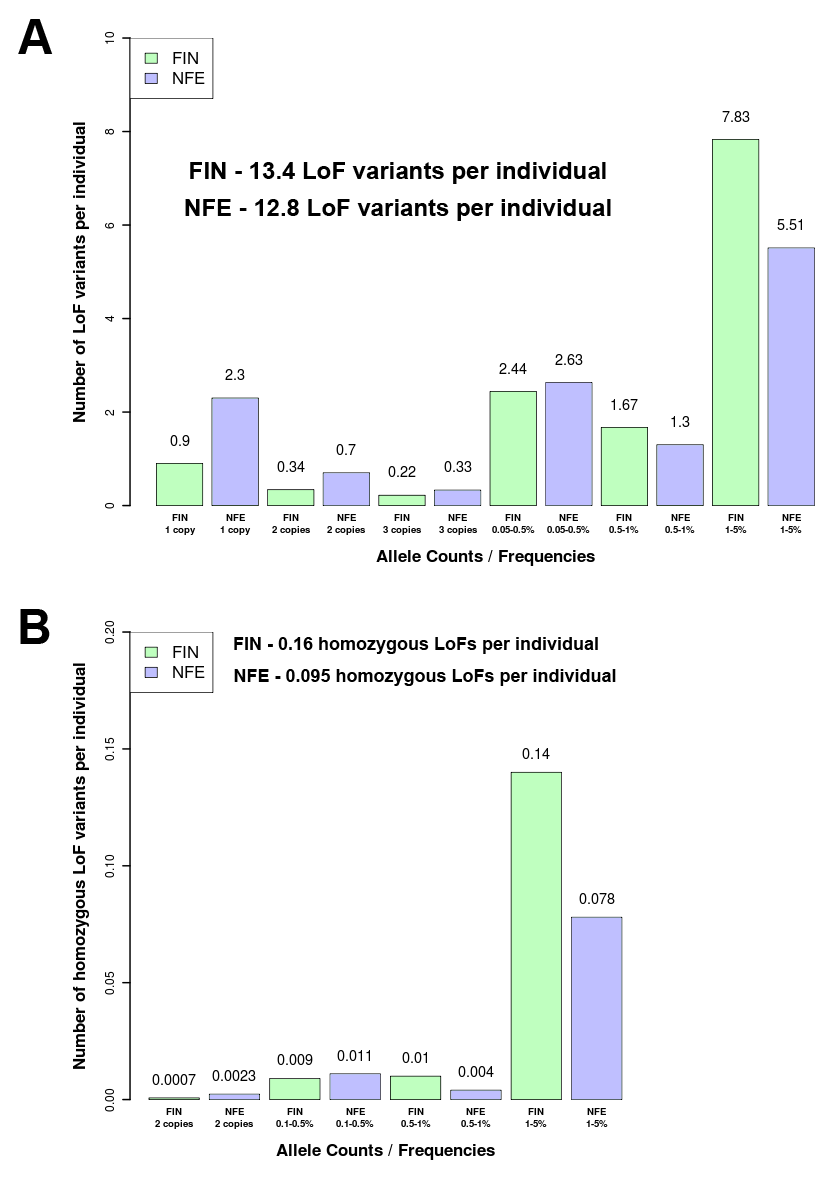

Supplement: Figure S3 — Distribution of LoF variants per individual. (A) Number of LoF variants in an average Finn vs NFE individual. (B) Number of homozygous LoF variants in Finns vs NFEs per individual. (DOCX) [file pgen.1004494.s003.docx]

Figure S4


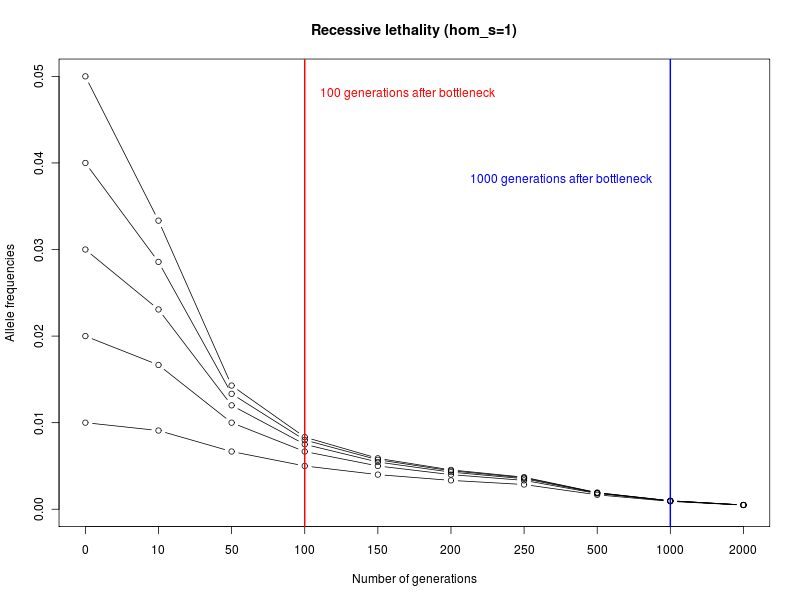

Supplement: Figure S4 — Simulations for a set of variants (ranging from 1% to 5% allele frequencies) with complete recessive lethality. The red line indicates the expected allele frequencies in present-day Finns (where the Finnish bottleneck occurred ∼100 generations ago) and the blue line indicates the expected allele frequencies in Finns 1,000 generations after the Finnish bottleneck, similar to the out-of-Africa bottleneck which occurred >1,000 generations ago. (DOCX) [file pgen.1004494.s004.docx]

Figure S5

| 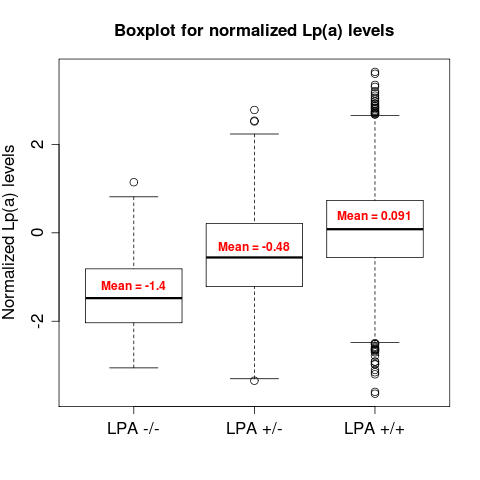 | 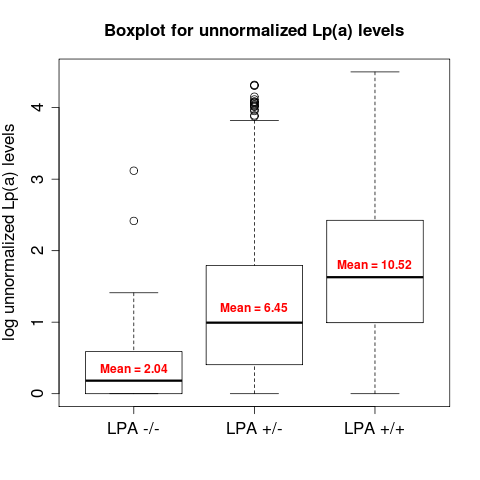 |
| --- | --- |
| 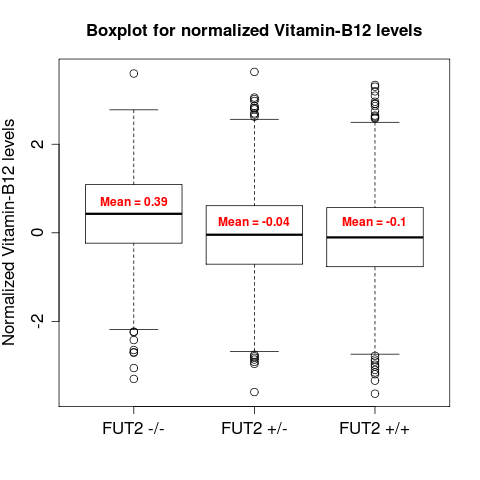 | 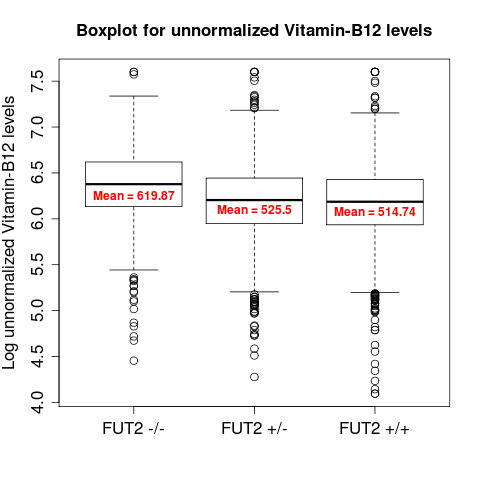 |
| 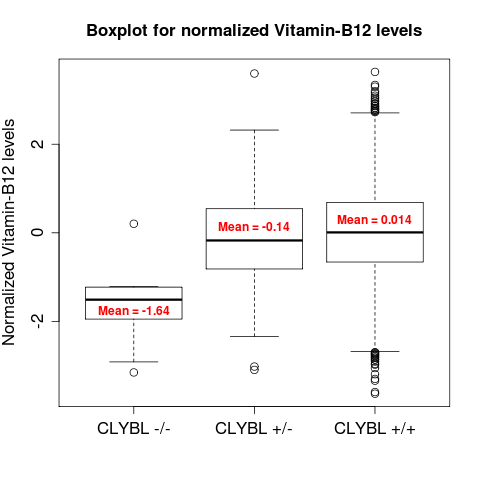 | 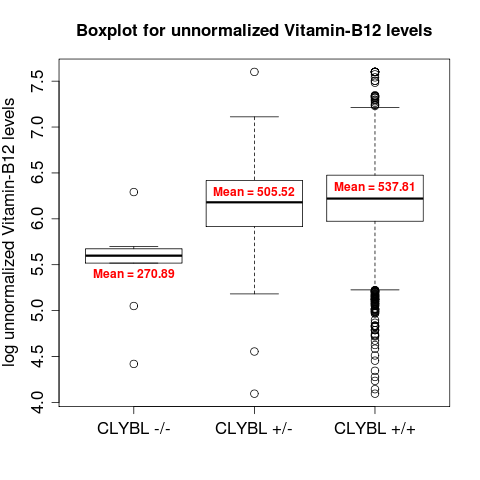 |
| 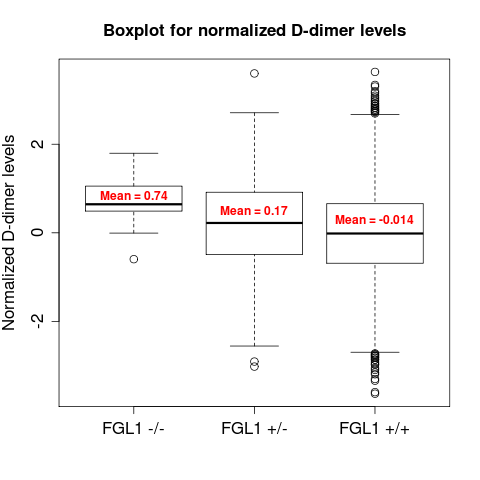 | 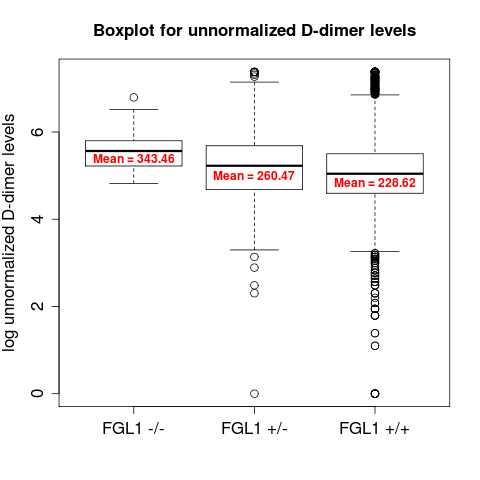 |
| 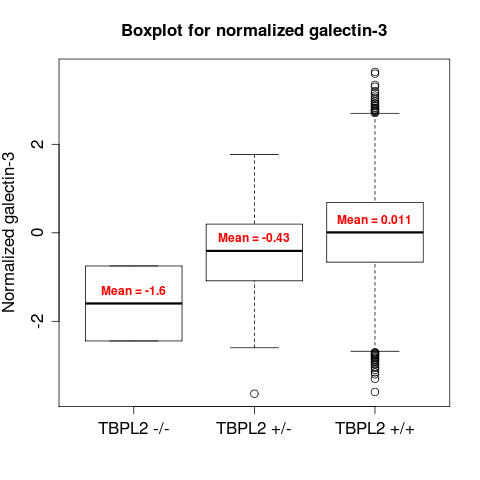 | 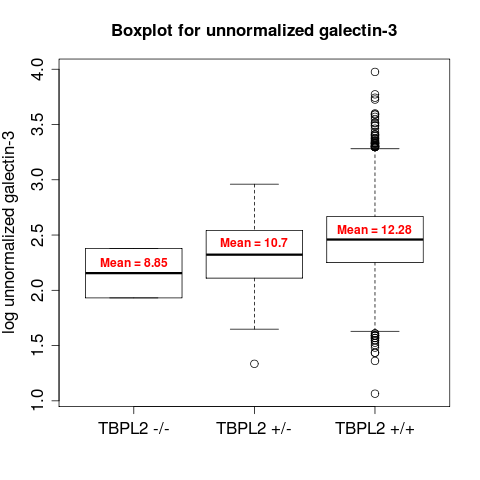 |
| 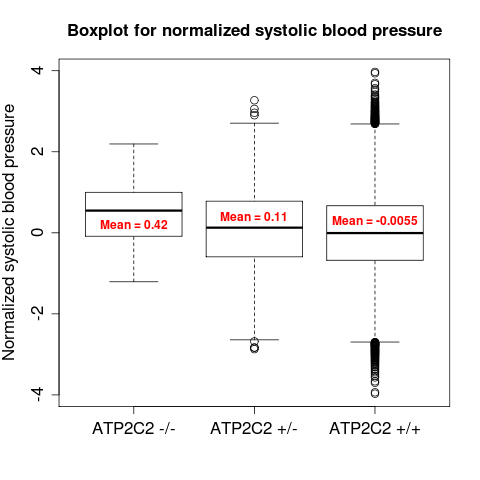 | 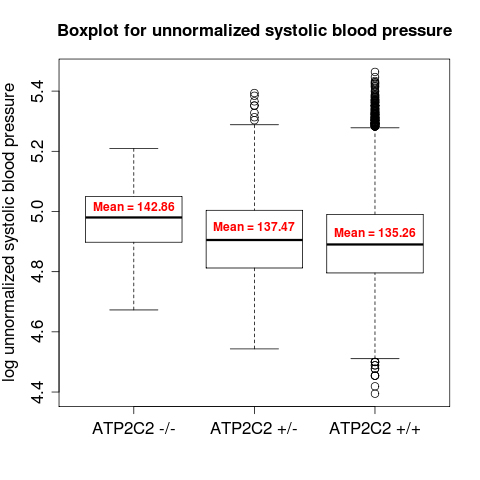 |
| 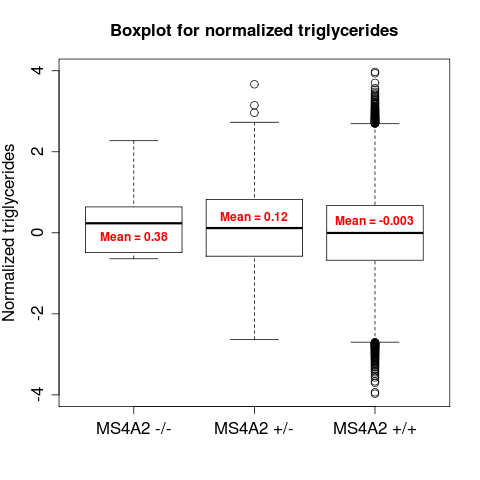 | 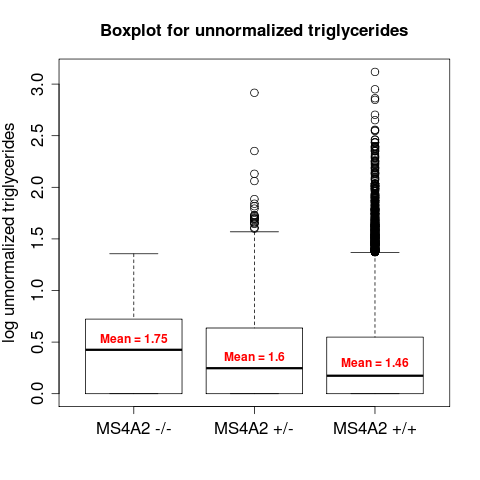 |

Supplement: Figure S5 — Boxplots for the known and novel associations. (DOCX) [file pgen.1004494.s005.docx]

Figure S6


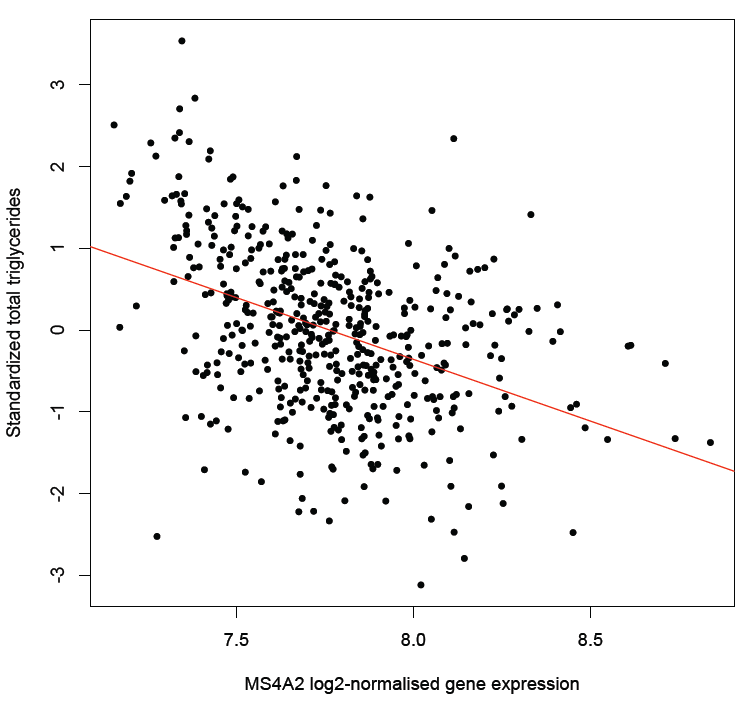

Supplement: Figure S6 — Correlation between triglycerides and MS4A2 gene expression. (DOCX) [file pgen.1004494.s006.docx]
